# Optimal multisensory integration leads to optimal time estimation

**DOI:** 10.1038/s41598-018-31468-5

**Published:** 2018-08-30

**Authors:** Yuki Murai, Yuko Yotsumoto

**Affiliations:** 1 Department of Psychology, University of California, Berkeley, USA; 2 Japan Society for the Promotion of Science, Tokyo, Japan; 3 Department of Life Sciences, The University of Tokyo, Tokyo, Japan

## Abstract

Our brain compensates sensory uncertainty by combining multisensory information derived from an event, and by integrating the current sensory signal with the prior knowledge about the statistical structure of previous events. There is growing evidence that both strategies are statistically optimal; however, how these two stages of information integration interact and shape an optimal percept remains an open question. In the present study, we investigated the perception of time as an amodal perceptual attribute. The central tendency, a phenomenon of biasing the current percept toward previous stimuli, is used to quantify and model how the prior information affects the current timing behavior. We measured the timing sensitivity and the central tendency for unisensory and multisensory stimuli with sensory uncertainty systematically manipulated by adding noise. Psychophysical results demonstrate that the central tendency increases as the uncertainty increases, and that the multisensory timing improves both the timing sensitivity and the central tendency bias compared to the unisensory timing. Computational models indicate that the optimal multisensory integration precedes the optimal integration of prior information causing the central tendency. Our findings suggest that our brain incorporates the multisensory information and prior knowledge in a statistically optimal manner to realize precise and accurate timing behavior.

## Introduction

From speech generation to music performance, many sensorimotor behaviors in daily life require accurate coding of event duration^1^. However, sensory information available for organisms is sometimes too noisy and impoverished to perceive the world as stable, and to make appropriate decisions^2^. Indeed, many studies have demonstrated that our brain utilizes various cues to estimate duration^3–6^. In particular, a considerable number of researchers have investigated how the perception of duration relies on information integration from multiple signal sources^7^.

One strategy in compensating for sensory uncertainties is to integrate redundant sensory information^8^. Since events in the outer world often generate multiple sensory signals, the timing system can estimate duration efficiently by collating information from different sensory modalities^9,10^.

In addition to the information redundancy occurring from one same event, the timing system can also utilize a statistical structure of previous events. The central tendency is a representative example of how the past temporal information has an impact on the current time perception: the percept of current stimulus duration is biased towards the mean of the previously presented durations^11,12^. The central tendency suggests that our brain stores the past information and biases the current sensory input under the assumption that the current event is likely to have similar characteristics with the statistical patterns of previous events.

Previous studies have demonstrated that our brain adopts a statistically optimal strategy both for the multisensory integration^13,14^ and the central tendency^15,16^. A stimulus feature defined by multiple sensory signals is optimally estimated by taking a weighted average of the unisensory estimates. For example, the visual information is more weighted than the auditory or haptic information to perceive stimulus location because vision has a higher spatial sensitivity than audition or touch. However, if the visual information is degraded by blurring stimulus or by adding noise, the weights of other modalities increase^13,14^. That is, multisensory signals are integrated depending on how reliable each modality’s information is. Similarly, the central tendency has been explained computationally from a Bayesian perspective^7^. The Bayesian model of time perception assumes that the timing system in the brain utilizes the distribution of previously presented durations as a prior information and estimates the current stimulus duration optimally by combining the prior information and the current sensory likelihood^15,17^. This Bayesian model predicts that the prior information has a larger effect on the time estimation when the current sensory input is unreliable. The computational strategies in the multisensory integration and in the central tendency have a common feature that the statistical optimality is obtained depending on the sensory uncertainty of events.

If the purpose of the timing behavior in daily life is to estimate time as accurately and precisely as possible, the optimal solution of time estimation should be to utilize as much information as possible. To this goal, the brain should somehow combine the prior information and the current sensory redundancy. However, it is highly debatable how different sources of temporal information interact and realize an optimal timing behavior. While conventional models of time perception assume the presence of a modality-independent central clock^18,19^, some researchers have suggested that the timing system in the brain is more distributed across local neural circuits and not necessarily integrated optimally. Heron *et al*. (2013) suggested that an adaptation-based hysteretic effect of time perception precedes multisensory integration^20^. Burr *et al*. (2009) demonstrated that the auditory modality dominates time perception over the visual modality, and that the multisensory integration of time is suboptimal^21^. On the other hand, other researchers have advocated a modality-independent central process contributing to time perception and its prior formation. Roach *et al*. (2017) suggested that the prior information can be generalized across sensory modalities^22^. Stauffer *et al*. (2012) proposed a hierarchical structure of modality-dependent and modality-independent temporal processing^23^.

**Figure 1:**
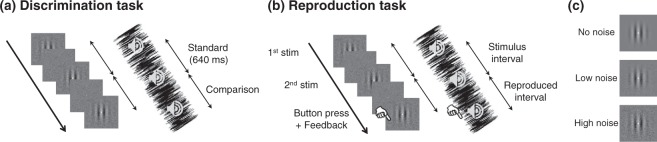
Schematics of the experiments. (**a**) Experimental procedure of the duration discrimination task. Three brief flashes or tones were sequentially presented, and subjects judged whether the first was longer or shorter than the second. The order of standard and comparison stimuli was counter-balanced. (**b**) Experimental procedure of the duration reproduction task. Two brief flashes or tones were sequentially presented, and subjects pressed buttons to reproduce the duration. A sensory feedback was presented immediately after the subjects’ responses. (**c**) An example of noise manipulation for visual stimuli. The background noise intensity was 0.66 (High), 0.22 (Low) or 0 (No) times the threshold noise level measured in the detection threshold task (see Methods section).

Previous studies have not directly investigated the central tendency for multisensory stimuli. Thus, how the sensory redundancy and the prior information are integrated to realize an optimal timing behavior still remains unclear. The present study aims to elucidate how the multisensory integration affects the timing sensitivity and the bias resulting from the central tendency. We systematically manipulated the sensory uncertainty for unisensory and multisensory stimuli by adding sensory noise, and examined how the sensory uncertainty in each modality impacts the multisensory timing sensitivity and bias. The experiment consisted of two tasks: the duration discrimination task, and the duration reproduction task. We measured the timing sensitivity by the duration discrimination task (Fig. 1a) and the central tendency by the duration reproduction task (Fig. 1b) with the signal-to-noise ratio manipulated across modalities by using the threshold values obtained in the preliminary detection threshold experiment (Fig. 1c). The behavioral data and our computational model demonstrate that two sources of contextual information –prior information and sensory redundancy– are optimally integrated.

## Results

### Sensory uncertainty impairs sensitivity and increases bias

**Figure 2:**
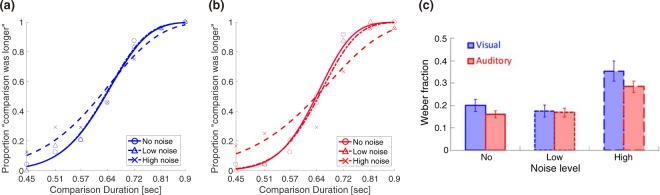
Sensory uncertainty impairs timing sensitivity. Psychometric functions of visual (**a**) and auditory (**b**) durations for a typical subject. The probabilities at which the subject judged that the comparison (450–900 ms) was longer than the standard (640 ms) were plotted, and fitted to the psychometric function. The discrimination sensitivity was defined by the Weber fraction, which is the ratio of the just noticeable difference (JND) to the standard. (**c**) Group results of Weber fractions for visual and auditory unisensory timing across modalities and different noise levels. Error bars indicate the standard error of the mean (SEM).

We first examined how the sensory uncertainty affects the timing sensitivity for unisensory stimuli. The timing sensitivity was measured by fitting the cumulative normal function to the data of the discrimination task (Fig. 2a,b) and calculating the Weber fraction. For both visual and auditory stimuli, the Weber fraction increased as the noise intensity increased (Fig. 2c). A two-way repeated-measures ANOVA, with noise level (high, low, or no) and sensory modality (visual or auditory) as factors, revealed a significant main effect of noise level (F (2,36) = 17.5, p < 0.001) but no significant main effect of stimulus modality (F (1,36) = 1.99, p = 0.17) and no significant interaction (F (2,36) = 0.42, p = 0.66). This result indicates that a larger sensory noise impairs the timing sensitivity in both modalities.

**Figure 3:**
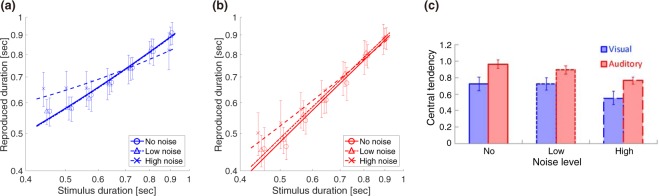
Sensory uncertainty increases central tendency. A typical subject’s data for visual (**a**) and auditory (**b**) stimuli. To quantify the central tendency, reproduced durations are linearly fitted to stimulus durations. A smaller slope value means a larger central tendency. Each dot represents reproduced duration in one trial. (**c**) Group results of the central tendency across modalities and noise levels. The regression slope smaller than 1 means the central tendency occurred. Error bars indicate SEM.

We next quantified the central tendency in the reproduction task by linearly regressing the reproduced duration to the stimulus duration (Fig. 3a,b). The slopes of the linear regressions were compared across conditions as indices of the central tendency. For example, if stimulus durations were reproduced veridically then the slope should be 1. However, if the central tendency occurred and the duration estimates were biased toward the mean, the slope value should be smaller than 1. Along with the decrement in sensitivity for noisier stimuli, the central tendency bias increased for such stimuli. For both visual and auditory stimuli, the regression slope decreased as the noise intensity increased (Fig. 3c). A two-way repeated-measures ANOVA, with noise level (high, low, no) and sensory modality (visual or auditory) as factors, revealed significant main effects of noise level (F (2,36) = 6.2, p = 0.005) and stimulus modality (F (1,36) = 17.35, p < 0.001), and no significant interaction (F (2,36) = 0.19, p = 0.83). Consistent with previous studies, the central tendency was weaker in the auditory domain than in the visual domain^16,24^.

Although the reproduction error we focus in the present study is the central tendency bias, it has also been reported that subjects overestimate or underestimate stimulus durations overall due to various modality-dependent effects^7^. In a further analysis, we estimated the overall reproduction error by calculating the difference between the mean of reproduced durations and that of stimulus durations. If the central tendency is the only source of reproduction bias, the grand mean of reproduced durations should be equal to the physical mean of stimulus durations, regardless of the central tendency. In actuality, the visual durations were significantly overestimated (Supplementary Fig. 1). A two-way repeated-measures ANOVA, with noise level (high, low, no) and sensory modality (visual or auditory) as factors, revealed a significant main effect of noise level (F (2,36) = 12.3, p = 0.001) and stimulus modality (F (1,36) = 66.3, p < 0.001), and no significant interaction (F (2,36) < 0.02, p = 0.98).

### Multisensory integration improves sensitivity and decreases bias

**Figure 4:**
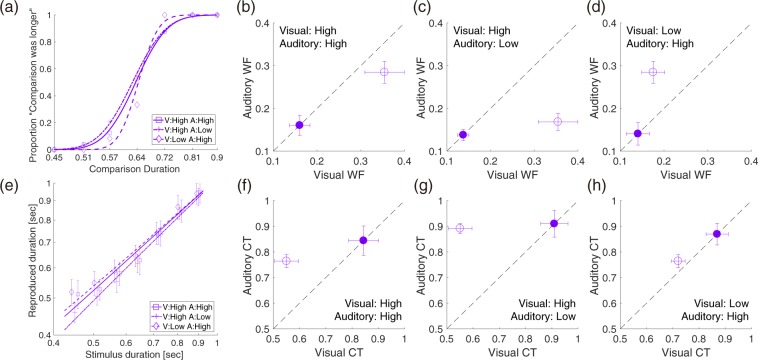
Unisensory and multisensory Weber fractions (WF; (**a**–**d**)) and central tendencies (CT; (**e**–**h**)). A typical subject’s psychometric functions (**a**) and reproduction data (**e**) for audiovisual stimuli. Legends in each panel show a combination of visual and auditory noise levels: (**b**,**f**), high visual noise and low auditory noise (**c**,**g**), and low visual noise and high auditory noise (**d**,**h**). Open circles denote unisensory WFs and CTs replotted from Figs 2c and 3c. Filled circles denote WFs and CTs for audiovisual stimuli. Error bars indicate SEM.

How does the multisensory integration affect the timing sensitivity and the central tendency? To examine how the sensory uncertainty in each modality affects the multisensory timing behavior, we measured the timing sensitivity and the central tendency for three combinations of visual and auditory noise levels: high visual and high auditory noise, high visual and low auditory noise, and low visual and high auditory noise. We used these three conditions to observe the timing behavior when two sensory information are equivalently noisy, or one modality is more reliable. Figure 4a–d show the audiovisual timing sensitivities for these three conditions (high visual and high auditory noise in Fig. 4b; high visual and low auditory noise in Fig. 4c; low visual and high auditory noise in Fig. 4d) in relation to the unisensory timing sensitivity. The multisensory Weber fraction was significantly lower than the lower Weber fraction of the two modalities in all of three conditions (p < 0.001, Cohen’s d = 2.67 for Fig. 4b; p = 0.028, d = 0.86 for Fig. 4c; p < 0.001, d = 1.30 for Fig. 4d; p-values were obtained by bootstrap tests with Bonferroni-correction for three comparisons). When the timing sensitivities between two modalities differed substantially (Fig. 4c,d), the audiovisual sensitivity was closer to the sensitivity for more reliable (less noisy) unisensory timing.

Figure 4e–h similarly show the audiovisual central tendencies for high visual and high auditory noise in Fig. 4f, high visual and low auditory noise in Fig. 4g, and low visual and high auditory noise in Fig. 4h. Along with the improvement in sensitivity, the audiovisual timing decreased the bias due to the central tendency. The multisensory central tendency was significantly smaller than the smaller central tendency of the two modalities in all of three conditions (p < 0.001, d = 2.29 for Fig. 4f; p = 0.004, d = 1.01 for Fig. 4g; p < 0.001, d = 1.40 for Fig. 4h; p-values were obtained by bootstrap tests with Bonferroni-correction for three comparisons). When the central tendencies between two modalities differed substantially (i.e., high visual and low auditory noise in Fig. 4g), the decrement in the central tendency was smaller than when the central tendencies between the two modalities were comparable.

The overall reproduction biases were fairly small for audiovisual stimuli (Supplementary Fig. 2), and no significant deviations from the physical mean of stimulus durations were observed in all conditions (p = 0.50 for Fig. S2a; p = 0.99 for Fig. S2b; p = 0.63 for Fig. S2c; p-values were obtained by bootstrap tests with Bonferroni-correction for three comparisons).

### Optimal multisensory integration in timing sensitivity

Such patterns of multisensory integration could be computationally accounted for in terms of statistical optimality. Some studies have suggested that the multisensory estimate of a given sensory feature (e.g., location, size) is integrated in a statistically optimal manner, that is called the maximum likelihood estimation, by taking a weighted average of unisensory estimates^13,14^. When visual and auditory signals are integrated, the audiovisual estimate is given as the following equation:

1 $${S}_{AV}={w}_{A}{S}_{A}+{w}_{V}{S}_{V}$$

where *S* denotes the unisensory or multisensory estimate of the sensory feature, and *w* denotes the weighting coefficient. The weighting coefficient for each sensory modality is determined based on the reliability (sensitivity) of sensory signals:

2 $${w}_{A}=\frac{{\sigma }_{A}^{-2}}{{\sigma }_{A}^{-2}+{\sigma }_{V}^{-2}},\,{w}_{V}=\frac{{\sigma }_{V}^{-2}}{{\sigma }_{A}^{-2}+{\sigma }_{V}^{-2}}$$

where *σ*^2^ represents the variance of the estimate. This integration strategy derives the smallest variance for the multisensory estimate, and is thus called “statistically optimal”:

3 $${\sigma }_{AV}^{-2}={\sigma }_{A}^{-2}+{\sigma }_{V}^{-2}$$

We tried to predict the timing sensitivity for audiovisual stimuli from the timing sensitivity for visual and auditory unisensory stimuli. In the present study, the timing sensitivity was obtained as Weber fractions that are proportional to the standard deviation of psychometric function. Thus, the optimal Weber fraction for multimodal timing is given by transforming the equation (3):

4 $$W{F}_{AV}^{2}=\frac{W{F}_{V}^{2}W{F}_{A}^{2}}{W{F}_{V}^{2}+W{F}_{A}^{2}}\le \,\min (W{F}_{V}^{2},W{F}_{A}^{2})\cdot $$

**Figure 5:**
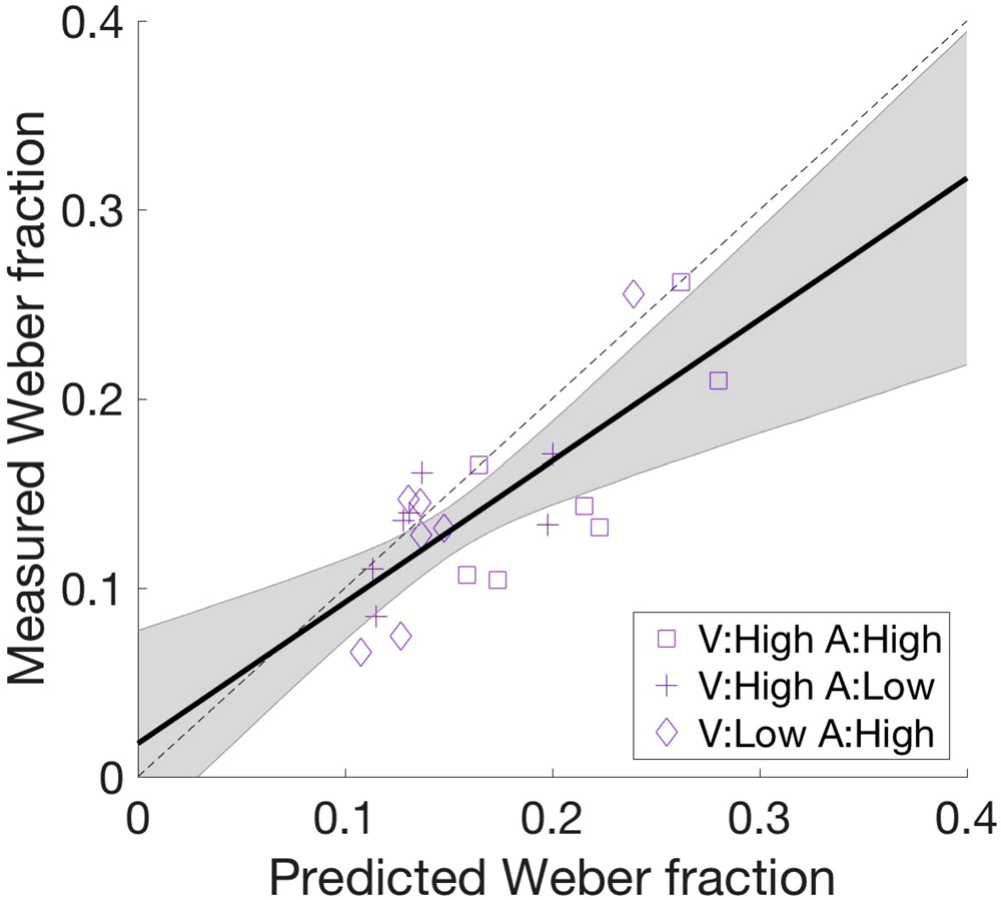
Measured and modeled audiovisual Weber fractions. Symbols indicate the combination of auditory and visual noise levels: high visual and high auditory noise (purple), low visual and high auditory noise (blue), and high visual and low auditory noise (magenta). The solid line indicates the linear fit of the measured Weber fraction to the predicted one. The shaded region indicates the 95% confidence interval of the linear fit.

This equation means that the optimal multisensory integration predicts the improvement of timing sensitivity for multisensory stimuli compared to the unisensory stimuli, which is exactly what we found. Consistent with previous studies^9,21^, the multisensory Weber fraction was smaller than the unisensory Weber fraction, as described in the previous section. Figure 5 compares the predicted Weber fractions calculated by the above equation and the experimentally measured Weber fractions. The predicted and measured Weber fractions were significantly correlated (r = 0.70, p < 0.001), however, the slope of the best-fit linear regression was 0.70, suggesting that the measured Weber fractions were smaller than the computationally predicted ones, consistent with previous studies^9,21^.

### Optimal multisensory integration precedes optimal time estimation

**Figure 6:**
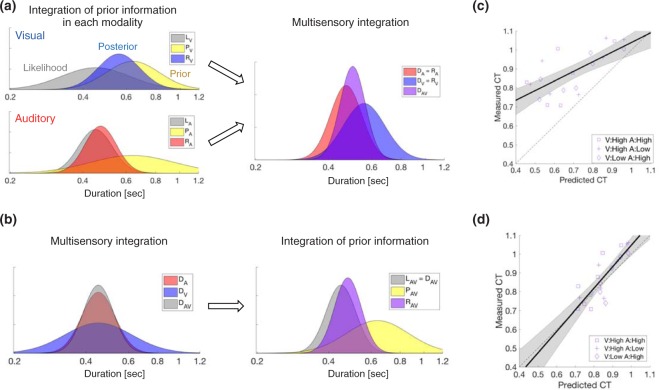
Two models of the interaction between the central tendency and the multisensory integration. (**a**) The first model assumes that the unisensory information is first biased by the prior integration, and then the biased unisensory representations are integrated by the maximum likelihood estimation. (**b**) The second model assumes that the unbiased unisensory representations are first integrated by the maximum likelihood estimation, and then the prior integration biases this multisensory representation of duration. Capitals in the legends represent the type of distribution: Likelihood (L), prior (P), posterior (R), and distributions in the multisensory integration (D). These graphs show examples for a stimulus duration of 0.45 sec, thus all the likelihood functions center at 0.45 sec. (**c**) Measured and modeled audiovisual central tendencies from the first model. (**d**) Measured and modeled audiovisual central tendencies from the second model. Symbols in (**c**) and (**d**) indicate the combination of auditory and visual noise levels: high visual and high auditory noise (purple), low visual and high auditory noise (blue), and high visual and low auditory noise (magenta). The solid line indicates the linear fit of the measured central tendency to the predicted central tendency. The shaded region indicates the 95% confidence interval of the linear fit.

The psychophysical results qualitatively demonstrated that the audiovisual presentation decreased the central tendency bias along with the improvement in sensitivity. An important question here is how the timing sensitivity affects the central tendency bias in relation to multisensory integration. Theoretically, there are two possibilities regarding how these two stages of information integration –the multisensory integration and the central tendency– interact and realize an optimal timing behavior. In the first model, the central tendency precedes the multisensory integration (Fig. 6a). This model assumes that the integration of prior information causing the central tendency occurs within each sensory processing, and then the duration estimates from both modalities are integrated by the maximum likelihood estimation. The second model, in contrast, assumes that the duration estimates from both modalities are first integrated, and then the integration of prior information occurs for this multisensory duration representation (Fig. 6b). To clarify the relationship between the multisensory integration and the central tendency, we compared the observed multisensory central tendency with the model predictions by these two models.

The first model assumes that the multisensory timing bias results from an optimal integration of unisensory biases. In the following models, we simulated all the distributions of duration representation by using a Gaussian distribution, according to previous studies^16,25^. The first stage of this model is the integration of prior information in each modality, which causes the central tendency in each sensory domain. We first estimated the posterior distributions in the unisensory timing from unisensory behavioral data. We assumed that both the prior and likelihood obey a Gaussian distribution with a mean and standard deviation (μ_P,_ σ_P_) and (μ_L,_ σ_L_). The prior distribution centers on the mean stimulus duration $\,\bar{{\rm{D}}}$ (μ_P_ = $\bar{{\rm{D}}}$), while the likelihood distribution for each stimulus duration centers on each physical stimulus duration. The width of the likelihood function was defined by the Weber fraction and the stimulus duration D:

5 $${\sigma }_{L}=WF\times D$$

According to Bayes’ rule, the posterior distribution for a given stimulus duration obeys a Gaussian distribution with the following mean:

6 $${\mu }_{R}={\mu }_{L}-\frac{{{\sigma }_{L}}^{2}t}{{{\sigma }_{P}}^{2}\,+\,{{\sigma }_{L}}^{2}}$$

where *t* represents the difference between the stimulus duration and the mean stimulus duration (t = ${\mu }_{L}-\,\bar{{\rm{D}}}$). Since the magnitude of central tendency was measured by the linear regression of the reproduced duration to the stimulus duration, its regression slope can be calculated from equation (6):

7 $$CT=\frac{{{\sigma }_{P}}^{2}}{{{\sigma }_{P}}^{2}+{{\sigma }_{L}}^{2}}$$

The width of unisensory prior function was estimated by assigning the observed values of the central tendency and the Weber fraction to equation (7). The posterior distribution for each stimulus duration was calculated by multiplying the likelihood function and the prior distribution described above. Then, these auditory and visual posterior distributions were integrated by the maximum likelihood estimation: equations (1) and (2). The model prediction of audiovisual central tendency was obtained by regressing the peaks of simulated audiovisual distributions for each stimulus duration to the stimulus durations. As shown in Fig. 6c, the predicted central tendency significantly overestimated the experimentally measured one (t (20) = 8.07, p < 0.001, Cohen’s d = 1.76).

The second model, in contrast, assumes that unbiased unisensory representations of duration are first integrated, and this multisensory representation is biased by the prior integration. The first stage of this model is the multisensory integration of auditory and visual input. Here, the unisensory duration representation is not biased yet, thus the peak of the unisensory duration distribution is the same as the stimulus duration. The width of each unisensory distribution was defined by the measured Weber fractions multiplied by the stimulus duration. These unisensory distributions were integrated by the maximum likelihood estimation, and this integrated multisensory distribution was used as a likelihood function in the prior integration. The peak of the multisensory prior function was defined as the mean stimulus duration $\,\bar{{\rm{D}}}$ (μ_P_ = $\bar{{\rm{D}}}$); The width of the multisensory prior function was defined as the average of the estimated auditory and visual prior widths, that are the same as the unisensory prior widths estimated in the first model. Instead of integrating the unisensory prior distributions by the maximum likelihood estimation, we adopted this distribution tentatively because this model assumes the prior representation emerges in a processing after information from both modalities are already integrated, not in the integration process of multiple sensory information. We will further examine actual prior distributions for the unisensory and multisensory timings in the next section. As described above, we simulated the likelihood and prior functions for multisensory stimuli from unisensory ones. The model prediction of audiovisual central tendency was obtained by assigning these simulated distribution widths to equation (7). As shown in Fig. 6d, the predicted central tendencies matched more accurately with the measured ones (t (20) = 1.61, p = 0.12, Cohen’s d = 0.35). These results suggest that the multisensory integration precedes the integration of prior information.

### Visual, auditory, and audiovisual timings utilize different prior information

The computational model revealed that the multisensory integration precedes the integration of prior information. Does such a Bayesian computation is implemented only in higher cognitive processing stages after the integration of multisensory information? Indeed, some researchers have suggested that the hysteresis-based perceptual misjudgments like the central tendency depend on higher cognitive functions such as memory and decision making^26^, and a recent psychophysical work also demonstrated that the prior information could be acquired across sensory modalities^22^. In contrast, other psychophysical studies have demonstrated that such computational strategies are also implemented in the early sensory processing^16,24^. To address this question, we further modeled how the prior information had an impact on the time estimation for unisensory and multisensory stimuli. If the higher cognitive processing is responsible for all the unisensory and multisensory timings, the same prior representation should be involved in timing for all the unisensory and multisensory stimuli.

In comparison with using a true stimulus distribution (e.g., a uniform distribution within the range of stimulus duration) as the prior, the Gaussian prior used in this paper has the advantage of being able to evaluate the internal representation of prior distribution by changing the width parameter. A wide prior width means the prior was close to a uniform distribution, that is, the prior information had only a little impact on timing behavior and the sensory likelihood determined timing behavior.

**Figure 7:**
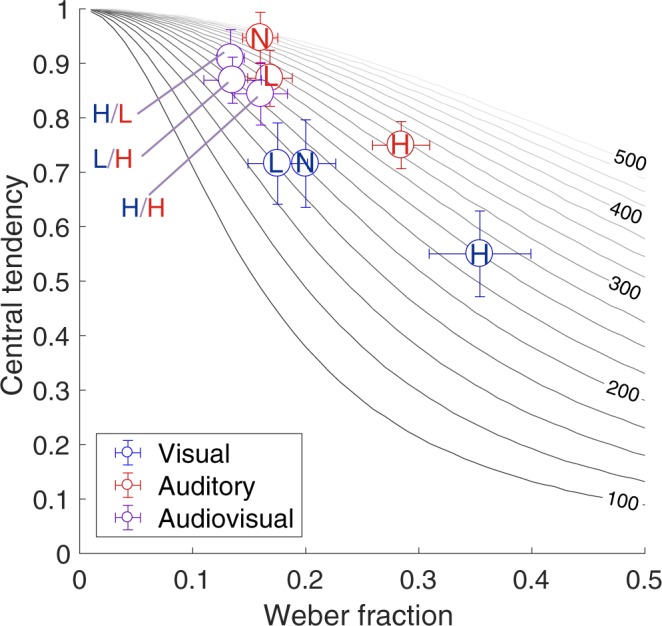
Relationship between the central tendency and the Weber fraction. The magnitude of the central tendency (smaller values mean larger central tendency) is plotted against the Weber fraction. The curves represent predictions of computational models with different widths of the prior (100 ms for the darkest and 500 ms for the lightest curve). Color indicates stimulus modality. Each dot represents the data in each noise level, and the noise levels are indicated as L (low) and H (high). Error bars indicate SEM.

Instead of using the observed Weber fraction and central tendency values as described in the previous section, we simulated the magnitude of central tendency for different prior widths and Weber fractions from equation (7). Figure 7 shows how the central tendency covaries with the Weber fraction. There was a general tendency that the central tendency increased as the Weber fraction increased, as predicted from the Bayesian model. At the same time, this graph demonstrates that the prior width varied substantially across sensory modalities even if the timing sensitivities were similar. For example, while the Weber fractions for the visual stimuli with low noise, the auditory stimuli with low noise, and the audiovisual stimuli with high visual and high auditory noise were comparable (~0.17), the estimated prior widths for these three conditions were 177, 282, and 238 ms, respectively. The wider prior width in the auditory modality suggests that the auditory timing is more weighted on the sensory likelihood and less on the prior information than the visual timing.

## Discussion

In the present study, we found that the sensory uncertainty impairs the timing sensitivity and amplifies the central tendency. These results are consistent with a Bayesian prediction: the noisier the sensory representation, the larger the central tendency. Cicchini *et al*.^16^ demonstrated this relationship by showing that an individual with high timing sensitivity is less susceptible to the central tendency. The present study, on the other hand, clarified that this statement also holds true within-individually. Importantly, our results demonstrated such prior information is also utilized in the auditory domain, not only in the visual domain. A couple of studies reported that the auditory modality shows no or only a very weak central tendency compared to the visual modality^16,24^, implying that the auditory timing utilizes only the sensory likelihood, and rarely the prior information. The present study challenged this view by demonstrating that the auditory timing is also affected by the prior information when the sensory signal is ambiguous. Previous studies have used clear and unambiguous sound stimuli like the “no noise” condition in our study, which would lead to the failure of finding a robust central tendency in the auditory domain.

We further demonstrated that the multisensory integration improves both the timing sensitivity and the central tendency. With regard to the timing sensitivity, previous studies have shown apparently contradictory results. The optimal integration of multiple sensory signals predicts an improvement in timing sensitivity. However, this prediction has not been confirmed experimentally in some studies^21,27^. Hartcher-O’Brien *et al*. (2014) argued that the studies which reported a sub-optimal integration used “empty intervals” that are defined by two brief stimuli and have no sensory input during the duration to be timed, and suggested that the multisensory information is optimally integrated only when durations are defined by “filled intervals” that the sensory signal is present throughout the duration^9^. In the present study, noise stimuli were always presented between two brief target stimuli defining a duration, thus our stimuli could be regarded as filled intervals, which might facilitate the optimal multisensory integration. Another possible reason why some studies failed to observe the optimal multisensory integration could be the difference in timing sensitivity between modalities. The optimal integration is more effective when the timing sensitivities from multiple signals are comparable. Otherwise, the integrated estimate should rely heavily on the most reliable unisensory signal, and the advantage of integration diminishes. In the studies that reported the sub-optimal multisensory integration, one of the signal sources was much more sensitive than the other signals, thus the effect size of the improvement would be too small to reach statistical significance.

Consistent with previous studies, we did not find a significant difference in timing sensitivity for empty intervals between modalities^24,28^. Nevertheless, the central tendency varied greatly for auditory and visual stimuli. These results suggest that the difference in the central tendency between modalities cannot be explained by the difference in the sensory uncertainty, but by how each sensory modality exploits the prior information. Indeed, the computational model suggested that the visual, auditory and audiovisual durations are estimated based on different prior information.

A recent psychophysical study demonstrated that one can acquire a single prior representation if the visual and auditory stimuli are alternately presented within a session, and suggested that the prior information could be generalized across sensory modalities^22^. In contrast, the present study demonstrated that the prior representation could be different across modalities if the visual and auditory stimuli are presented in different sessions, and that the central tendency for multisensory stimuli results from the optimal integration of unisensory biases. Our results suggest that our timing system does not necessarily use a general modality-independent prior representation irrespective of sensory input. Rather, purposive prior could be selected adaptively in conformity with the incoming sensory signals. Such an adaptive selection of prior may be efficient for updating the environmental information. If an environment contains multiple sources of signal, the prior representation should be generalized across various sensory inputs; if the signal source is rather limited and constant, a prior specific for the input would be more efficient.

Since the reproduction task requires motor responses, one might argue that the timing sensitivity in the reproduction task could be independent from the purely perceptual sensitivity measured in the discrimination task. However, past studies have demonstrated that perceptual and sensorimotor timing sensitivities are significantly correlated^29,30^. Furthermore, the fact that the audiovisual central tendency measured in the motor task can be successfully modeled by using the timing sensitivities measured in the perceptual task obviously suggests that the perceptual and sensorimotor timing share a common mechanism.

However, this does not necessarily mean that the perceptual and sensorimotor timing share the completely same timing system. Certainly, both the multisensory timing sensitivity and bias can be modeled with weights determined by unisensory sensitivities, but the model underestimated the improvements in the Weber fraction, consistent with a previous study^9^. One possible reason is that the timing sensitivity in the discrimination task could be overestimated by the decision uncertainty related to the binary decision process. Since the measured Weber fractions are not determined only by purely sensory processes, but also by post-perceptual processes, the sensory likelihood distribution could have a narrower tuning than that estimated from the obtained Weber fraction. Integrating visual and auditory Weber fractions may include decision-related noise distributions from two modalities redundantly, which would lead to the overestimation of the modeled multisensory sensitivity. It also should be noted that the sensorimotor timing might be able to exploit the timing noise inherent in the motor system. A recent psychophysical study reported that subjects reproduce auditory durations more precisely than visual durations^31^, in contrast to the fact that the perceptual discriminability was comparable between auditory and visual modalities. Although the present and previous studies have used the perceptual Weber fraction to estimate the likelihood function, further study will need to address how perceptual and motor noise contribute to the likelihood estimation.

In addition to the width of likelihood distribution defined by the Weber fraction, one should also be cautious about the peak of likelihood and prior distributions since the internal representation of stimulus duration could be biased overall compared to the physical stimulus duration. Past Bayesian modeling studies have assumed that both the likelihood and the prior are unbiased^16,25^, that is, centered on the physical stimulus duration and the physical mean of stimulus durations, respectively. However, these assumptions cannot account for the overall reproduction bias. Although previous studies have suggested that subjects can utilize the veridical (unbiased) stimulus distribution as the prior^17^, and the overall bias is an idiosyncratic bias independent from the central tendency^16^, no studies have directly investigated how such overall biases occur in relation to the Bayesian time estimation. Any stage of duration processing could be susceptible to such biases, including sensory encoding (likelihood), integration of previous information (prior), processes independent from these Bayesian estimations, which could potentially affect the model predictions.

Another outstanding question related to the sensorimotor timing is whether such a Bayesian computation is implemented in the sensory encoding stage or the decoding (motor production) stage. The present study demonstrated that the prior representation emerges adaptively depending on incoming sensory input, suggesting the presence of Bayesian mechanisms embedded in the sensory encoding processes. Consistent with this view, a recent fMRI study suggested that the brain activities associated with the central tendency occur during the stimulus encoding phase^32^, and psychophysical studies have reported similar Bayesian phenomena of time perception in perceptual tasks such as the duration bisection task^33^ and the duration discrimination task^34^. In contrast, several studies have suggested that a Bayesian time estimation is also associated with higher cognitive processes such as memory and decision-making^33–35^. Furthermore, various studies have shown that the motor learning in the spatial domain occurs in a Bayesian manner^36,37^, and it would be plausible that the motor system applies a similar Bayesian strategy also in the temporal domain. Since both perceptual and motor systems have inherent noise, it might be efficient to utilize both noise information to estimate time as accurately and precisely as possible.

Although the present study focused on the sensorimotor timing, the central tendency has been reported widely across various sensory and motor features including stimulus length^38^, stimulus size, numerosity^39^, and movement distance^40^. Furthermore, there is growing evidence that low-level sensory features such as orientation or motion direction are also attracted toward previously presented stimuli^41,42^. These lines of studies strongly support the view that similar statistical mechanisms are implemented at multiple sensory and motor processing stages. The present study suggests that the brain exploits the prior information stored at different processing stages adaptively in accordance with incoming sensory evidences.

## Methods

### Subjects

Seven healthy volunteers (4 males, 19–28 years old) participated in the experiment. All subjects reported to have normal hearing and normal or corrected-to-normal vision. This study was carried out in accordance with the recommendations of the ethics boards of the University of Tokyo with written informed consent from all subjects. All subjects gave written informed consent in accordance with the Declaration of Helsinki. The protocol was approved by the institutional review board of the University of Tokyo.

### Apparatus and stimuli

The auditory stimuli were presented through an Audio Stream Input/Output (ASIO) compliant USB audio interface (Roland UA-1G) and SONY MDR-XB500 headphones at 60 dB. The visual stimuli were presented on a CRT monitor (Mitsubishi Electric RDF223H, 1024 × 768 pixels, 120 Hz refresh rate). Subjects were seated 57.3 cm from the monitor in a dark soundproof room and the subjects’ heads were stabilized using a chin rest.

Stimuli were generated using MATLAB (MathWorks, R2014b) and the Psychophysics Toolbox^43,44^. We presented Gabor stimuli (0.5 cpd; SD of contrast envelope 1.6 deg; peak contrast 40%) as the visual target stimuli, and simple tones (600 Hz, 60 dB) as the auditory target stimuli. Each visual or auditory stimulus lasted for 20 ms. A cosine ramp of 5 ms was applied to the onset and offset of auditory targets. As noise stimuli, we presented dynamic random noise stimuli (8 × 8 deg) for visual noise, and white noise sound for auditory noise. The noise intensity (luminance contrast for visual noise; sound level for auditory noise) was calibrated for each participant by a preliminary experiment (see below). Sound levels were calibrated with a WS1361 sound-level meter (Wensn), and the synchronization between auditory and visual stimuli was verified using a Tektronix TBS 1042 oscilloscope with a photodiode and a microphone.

### Procedure

The experiment consisted of two tasks: the duration discrimination task, and the duration reproduction task. The order of the discrimination task and the reproduction task was randomized across subjects.

#### Pre-experimental calibration of noise intensity

To equalize the signal-to-noise ratio between the visual and auditory stimuli, we first measured the visual and auditory detection thresholds. In the experiment, the target was embedded in the stream of noise stimulus (dynamic random noise for vision; white noise sound for audition). Target stimuli appeared in half of all trials, and subjects answered whether the target was present or absent. The noise intensity (luminance contrast for vision; noise loudness for audition) was systematically manipulated and the correct response rate was calculated for each noise intensity. Psychometric functions were fitted to the data and we defined the detection threshold individually as the noise intensity at which the subject judged target presence correctly with a probability of 75%.

#### Duration discrimination task

The timing sensitivities for visual, auditory, and audiovisual stimuli were measured by the duration discrimination task. In this experiment, three successive flashes or tones that marked two neighboring durations were presented, and subjects reported whether the first duration was longer or shorter than the second (Fig. 1a). The standard duration was 640 ms; the comparison durations ranged from 450 ms to 900 ms, and were spaced logarithmically. The presentation order of the standard and comparison durations was counter-balanced. All comparison durations were presented 24 times.

The flashes or tones were embedded in noise stimuli. The noise presentation started 500–900 ms before the first flash/tone and ended 500–900 ms after the third flash/tone. The noise intensity was 0.66, 0.22 or 0 times the threshold noise level obtained in the detection threshold experiment (Fig. 1c). This manipulation aimed to equalize the signal-to-noise ratio between the visual and auditory stimuli. Hereinafter, we call these three noise levels as high, low, and no noise conditions.

All subjects completed three experimental blocks: visual, auditory, and audiovisual experiments. These three experiments were conducted in different experimental days, and the order of the experiments was randomized across subjects. In the audiovisual experiment, the visual and auditory stimuli were presented synchronously, and there were three combinations of visual and auditory noise levels: high visual and high auditory noise; high visual and low auditory noise; low visual and high auditory noise. These three combinations mean the equivalently noisy, the vision-noisier, and the audition-noisier conditions, respectively.

In the analyses, the probability at which the subject judged the comparison to be longer than the standard was plotted as a function of the comparison duration. A cumulative normal function was fitted to the data and we calculated the Weber fraction to quantify timing sensitivity (Fig. 2). Weber fractions were defined as the ratio of the standard deviation of the best-fit psychometric function to the standard duration (σ/640 ms).

#### Duration reproduction task

The magnitude of central tendency was measured separately for visual, auditory, and audiovisual stimuli by the duration reproduction task. In the experiment, a pair of stimuli was briefly presented, one after the other. Subjects were asked to reproduce the duration between the pair of stimuli by pressing a button. Immediately after the subject’s response, a sensory feedback (a flash or a tone) was given in every trial (Fig. 1b). The stimulus durations ranged from 450 ms to 900 ms, and were spaced logarithmically. Each duration was presented 60 times.

All subjects completed the visual, auditory and audiovisual experiments. The noise intensities in the experiments were the same as in the duration discrimination task. The order of the three experiments was randomized across subjects. At the beginning of each experiment, subjects completed a practice session with 50 trials in order to become accustomed to the task.

To prevent the influence of outlier trials, the data were excluded if the reproduced duration deviated more than 3 standard deviations from each condition’s mean. We quantified the central tendency by linearly regressing the reproduced duration to the stimulus duration. The slopes of the linear regressions were compared across conditions as indices of the central tendency.

### Data Accessibility

Data will be made available through Open Science Framework.

## Electronic supplementary material

Supplementary materials
