# Supplementary material for: Optimal multisensory integration leads to optimal time estimation
Source: Sci Rep. 2018 Aug 30;8:13068. doi: 10.1038/s41598-018-31468-5 (PMC6117357; doi:10.1038/s41598-018-31468-5)
Supplement: Supplementary file 1 — Supplementary materials [file 41598_2018_31468_MOESM1_ESM.docx]

Supplementary materials for “Optimal multisensory integration leads to optimal time estimation”

Yuki Murai^1,2,*^ and Yuko Yotsumoto^3^

^1^Department of Psychology, University of California, Berkeley, USA

^2^Japan Society of Promotion of Science, Tokyo, Japan

^3^Department of Life Sciences, The University of Tokyo, Tokyo, Japan

^*^corresponding author: ymurai@berkeley.edu


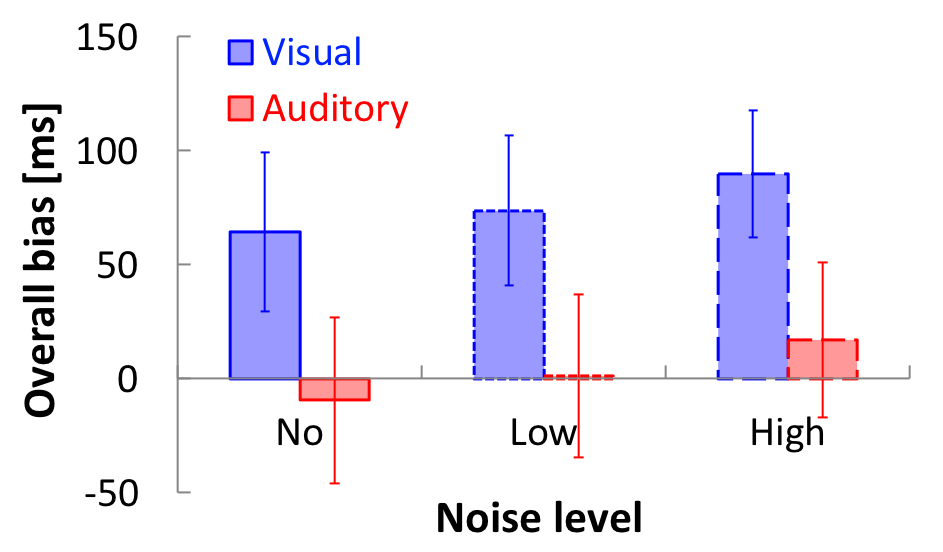
Supplementary Figure 1. Overall reproduction biases in unisensory timing. The overall reproduction bias was evaluated by calculating the difference between the mean of reproduced durations and that of stimulus durations. Error bars indicate the standard error of the mean (SEM).


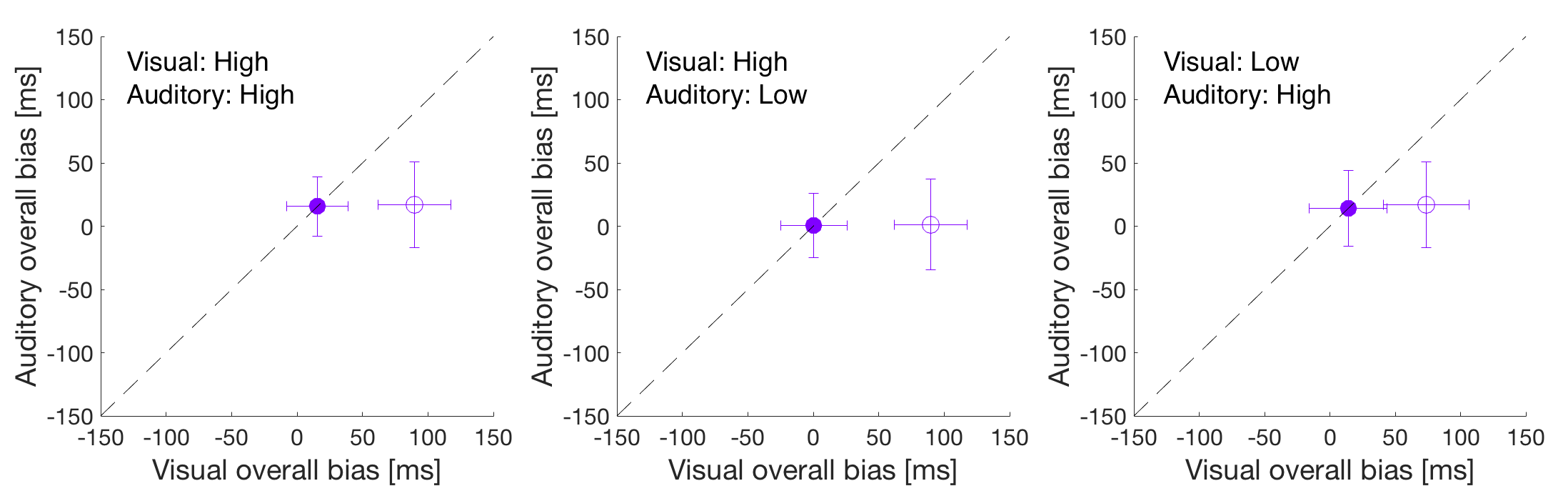


Supplementary Figure 2. Unisensory and multisensory overall reproduction biases. Legends in each panel show a combination of visual and auditory noise levels. Open circles denote unisensory reproduction biases replotted from Supplementary Figure 1. Filled circles denote reproduction biases for audiovisual stimuli. Error bars indicate SEM.
